# Determinants of acceptance of end-of-life interventions: a comparison between withdrawing life-prolonging treatment and euthanasia in Austria

**DOI:** 10.1186/s12910-015-0076-y

**Published:** 2015-12-01

**Authors:** Erwin Stolz, Franziska Großschädl, Hannes Mayerl, Éva Rásky, Wolfgang Freidl

**Affiliations:** Institute of Social Medicine and Epidemiology, Medical University of Graz, Universitätsstrasse 6/I, Graz, 8010 Austria; Institute of Nursing Science, Medical University of Graz, Billrothgasse 6, Graz, 8010 Austria

**Keywords:** Withdrawal of life-prolonging treatment, Euthanasia, Acceptance, Determinants

## Abstract

**Background:**

End-of-life decisions remain a hotly debated issue in many European countries and the acceptance in the general population can act as an important anchor point in these discussions. Previous studies on determinants of the acceptance of end-of-life interventions in the general population have not systematically assessed whether determinants differ between withdrawal of life-prolonging treatment (WLPT) and euthanasia (EUT).

**Methods:**

A large, representative survey of the Austrian adult population conducted in 2014 (*n* = 1,971) included items on WLPT and EUT. We constructed the following categorical outcome: (1) rejection of both WLPT and EUT, (2) approval of WLPT but rejection of EUT, and (3) approval of both WLPT and EUT. The influence of socio-demographics, personal experiences, and religious and socio-cultural orientations on the three levels of approval were assessed via multinomial logistic regression analysis.

**Results:**

Higher education and stronger socio-cultural liberal orientations increased the likelihood of approving both WLPT and EUT; personal experience with end-of-life care increased only the likelihood of approval of WLPT; and religiosity decreased approval of EUT only.

**Conclusion:**

This study found evidence for both shared (education, liberalism) and different (religiosity, care experiences) determinants for the acceptance of WLPT and EUT.

## Background

End-of-life decisions and particularly euthanasia are still controversially disputed in public and medical discourse in Europe, against the background of liberal legalisation in some Western European countries [1], increasing public acceptance [2], higher life expectancy and hereafter chronic suffering. Most discussions in this context, for which the acceptance in the general population can act as an anchor, tend to centre on euthanasia (EUT), i.e. on the voluntary hastening of death by having medical personnel deliberately administrate drugs with the intention to cause the patient’s death, whereas the deliberate withdrawal or withholding of treatment upon a patient’s voluntary request (WLPT) seems to be less controversial. Philosophically, there is a long-standing argumentation about a whether or not there is moral difference between WLPT and EUT [3–6]. Typically, those in favour of EUT claim that in principle and ceteris paribus, killing and letting die are not morally distinct, whereas opponents of EUT claim that these are indeed causally different and this difference in many situations entails moral significance. In contrast to the philosophical debate, differences between WLPT and EUT in legislation and daily medical practice are more unambiguous [7–10]. In Austria, for example, WLPT is legal if based on the patient’ will, whereas EUT is considered a culpable delict according to § 77 StGB, punishable with prison sentences ranging from six months to five years.

General acceptance in society, which is the focus of this article, also differs between WLPT and EUT. The gap between WLPT and EUT in popular approval – depending on national context and how questions are framed – ranges between 3 and 35 percentage points [11–17]. A recent survey from Germany [18], for example, found that 78 % of the population approved of WLPT compared to 67 % for EUT, and reported more undecided answers in the latter (20 %) compared to the former case (15 %). In this perspective, acceptance of end-of-life interventions can be considered as a three-tier model, starting with non-acceptance of end-of-life interventions, i.e. rejection of both WLPT and EU, approving WLPT as a first step, and approving of both WLPT *and* EUT as the second step.

Whereas overall acceptance levels in national societies for different forms and scenarios of end-of-life interventions have been documented, particularly comparisons between physicians, nurses and the general public are widespread [14, 15, 19], less is known about differences in determinants of different forms of end-of-life interventions. We wanted to find out, whether the same characteristics determine the approval or disapproval of these two different end-of-life interventions; more concretely, what characterises people who reject both forms of end-of-life interventions in contrast to those approving solely WLPT, and finally, to those who approve of EUT (and WLPT). The fact that research to date has shown only little interest in explicitly differentiating between determinants of approval between WLPT and EUT could be due to the widespread implicit assumption, that acceptance of both forms of end-of-life decisions are triggered by the same or a least similar set of determinants. The bulk of research on determinants of acceptance of end-of-life decisions in the general population has focused on approval of EUT (or physician-assisted suicide). This line of research has repeatedly identified religiosity as negative correlate and higher education and liberalism as positives correlates of EUT [20–27]. Furthermore, differences in acceptance between medical personnel, especially physicians, and the general population have also been outlined [14, 15, 19]. More recently, personal experiences regarding care for either seriously ill or dying persons were integrated as a potential factor of acceptance or rejection [24, 25, 28]. To the authors knowledge, only a handful of empirical studies assessed both WLPT and EUT at the same time [11, 12, 14, 15, 18]. Out of these, only two studies have statistically tested determinants separately for WLPT and EUT [14, 15], although the comparison represented only a secondary issue. Overall, these studies found men more approving of EUT compared to women, and vice versa regarding WLPT. The results regarding education were inconclusive. Furthermore, in these differentiating studies, being religious was associated only with acceptance for EUT but not for WLPT. The limiting factors of these studies are that they relied on a few (dichotomised) predictors or applied bivariate statistical tests only, and that they compared acceptance for WLPT and EUT separately, instead of explicitly linking these two forms of end-of-life decisions.

Based on these lacunae in the existing literature, this paper seeks to provide evidence whether or not the determinants of acceptance regarding WLPT and EUT differ in the general population. In order to assess whether the same or different determinants are relevant for each WLPT and EUT, we constructed a three-step outcome, differentiating between (1) those who reject both WLPT and EUT, (2) those, who approve WLPT while rejecting EUT, and lastly (3) those, who approve of both WLPT and EUT. We hypothesised that acceptance of EUT (and WLPT) would be subject to more polarisation than acceptance of WLPT alone, i.e. we expected more pronounced and stronger effects from known demographic, socio-economic, and attitudinal determinants. Particularly, we expected the level of religiosity to be of higher relevance for EUT than for WLPT alone, since EUT is explicitly rejected by religious authorities and belief systems (e.g. [29]) with reference to the ‘sanctity of life’. We expect attitudes towards withdrawing life-prolonging treatment to be generally less affected in this respect.

## Methods

### Study design

An omnibus survey representative of the Austrian adult population (<18 years) was conducted in early 2014. Stratified random sampling was used and target households were randomly drawn from the strata in proportion to the actual number of households. Selected households received a written invitation to participate and were contacted by phone in order to inform respondents about anonymity of all personal data and interview topics, and to obtain their consent for arranging computer-assisted personal interviews (CAPI). Within households, individuals were selected using the Kish-Selection-Grid, and again verbal informed consent was obtained from the selected participant on-site. Interviews were carried out by the Institute of Empirical Social Studies (IFES, Vienna) at the behest of the authors. The study was carried out in compliance with the principles laid down in the Helsinki Declaration. The conduct of this study was approved by the Ethics Committee of the Medical University of Graz (EK-number 26–425 ex 13/14). In total, 1,971 interviews were completed (response rate = 47.5 %).

### Variables

Acceptance of WLPT and EUT was measured by the following two items, which focused on the concrete action acceptable in both cases, and avoided the potentially misleading or confusing and emotionally charged term ‘euthanasia’ [13]. Answer categories included only ‘approve’ or ‘disapprove’ in order to encourage a definitive statement:

- WLPT: ‘Do you approve or disapprove that terminally ill and greatly suffering individuals have their wish to die fulfilled by withdrawing a medically-possible life-prolonging treatment?
- EUT: ‘Do you approve or disapprove that terminally ill and greatly suffering individuals have their wish to die fulfilled by a medical doctor administering a substance which causes their death?’

Based on these two dichotomous items, a multinomial outcome variable was created: rejecting both WLPT and EUT (1), approving of WLPT but rejecting EUT (2), and approving of WLPT and EUT (3). We combined the answers from the two binary variables into one multinomial outcome rather than to analyse them separately, since we wanted to portray the grading of different, though related levels of acceptance of interventions at the end of life, and especially to avoid amalgamation of those supporting WLPT in a heterogeneous group of respondents who support *only* WLPT and those who supported both WLPT *and* EUT. 16.7 % (330) of the respondents did not provide a valid answer (don't know or refuses) to any of the two dichotomous items. While more than 96 % of the respondents fell unambiguously into one of the three categories outlined above based on their their responses, 3.8 % (63) answered not according to expectation, i.e. they approved of EUT but not of WLPT, and were subsequently excluded from the analysis. In sum, this resulted in a total sample of *n* = 1,578.

The following predictor variables were included: socio-demographics – including sex, age (in years), household size (single, dual, 3+), highest level of education (compulsory school, apprenticeship or intermediate vocational degree, high school diploma, university) – subjective health status (1 = very good, 5 = very poor), and religious confession (Catholic, Protestant, Muslim, other, none). Respondents were also asked to specify whether they currently provide or had previously provided care to seriously ill (yes/no) or dying persons (yes/no), and whether they work in the health sector (physician, nurse, other). Finally, two attitudinal variables were included: self-reported liberalism (1 = strongly liberal, 4 = not at all liberal) and religiosity (1 = strongly religious, 4 = not at all religious). Age, health status, liberalism and religiosity were entered as continuous variables, and the remaining variables were dummy-coded. Missing values in the predictor variables (8.3 %) were multiply imputed (m = 5) using the bootstrap EM algorithm from package ‘Amelia’ (1.7.3, [30]) for R.

### Data analysis

All data analyses were performed using R: A language and environment for statistical computing (3.2.2) [31]. For bivariate statistics, Pearson’s *χ*^2^ test was used to assess relationships with categorical predictor variables. For continuous predictors, bivariate linear regression models were computed with each type of euthanasia as dummy variables in order to show statistically significant mean differences. Multinomial logistic regression (package ‘ZeligChoice’, 0.8-1, [32]) was used to model differences between the reference category (rejecting both WLPT and EUT) and approval of WLPT alone, and approval of EUT (and WLPT) across the imputed data-sets. Descriptive statistics were based on the first imputation.

## Results

### Descriptive and bivariate analysis

Characteristics of the final sample are shown in Table 1 (second column). Mean age was 49.7 years (SD = 16.3, range = 18–94), 53.2 % of the sample were female, and a majority had finished apprentice/vocational schooling as highest level of education (59.6 %). 73.3 % of the sample were Catholic and 17.2 % without confession. 30.9 % have had personal experience with caring for a seriously ill, and 29.6 % with a dying person. 9.9 % gave non-valid answers regarding WLPT, and 14.3 % regarding EUT. Among the valid answers, less than one quarter rejected WLPT and EUT (22.6 %), while about another quarter approved only of WLPT (23.1 %) and more than half (54.3 %) approved of both WLPT and EUT. Table 1 also shows the bivariate associations between the three outcome values and the predictor variables (columns 3–6). Significant bivariate differences exist for a majority of the predictor variables. Percentage differences regarding the approval of EUT (and WLPT) show, for example, between men and women, whereby the latter were less likely (50.5 %) to approve than the former (58.6 %). Furthermore, one third (33.0 %) of the respondents with compulsory education only rejected WLPT and EUT, as opposed to 22.4 % in High School leavers and only 10.5 % in those with a University degree. Respondents with experience in the care for seriously ill or dying individuals seemed more likely to approve of WLPT (28.5 % and 29.3 %) compared to those without such experience (20.7 % and 20.5 %). People without religious confession appeared notably more inclined to approve of both end-of-life interventions than to those belonging to religious denominations. Regarding religiosity, no bivariate difference exists between those who rejected both WLPT and EUT, and those who approved of WLPT alone. On the other hand, a clear bivariate difference was found between those rejecting both end-of-life interventions, and those approving of EUT and WLPT. Finally, liberal individuals were clearly more inclined to accept both WLPT and EUT.

**Table 1:** Sample characteristics and descriptive statistics

	Sample characteristics	Approval			
		None	WLPT only	EUT (and WLPT)	
Categorical Variables	N (%)	%	%	%	*χ* ^2^ *p*-value
Total sample	1,578 (100.0)	22.6	23.1	54.3	-
Gender					
Male	739 (46.8)	19.8	21.7	58.6	0.004
Female	839 (53.2)	25.0	24.4	50.5	
Education					
Compulsory school	206 (13.1)	33.0	19.4	47.6	<0.001
Apprentice/vocational	940 (59.6)	22.9	21.2	56.0	
High school diploma	232 (14.7)	22.4	25.4	52.2	
University	200 (12.7)	10.5	33.5	56.0	
Confession					
Catholic	1,157 (73.3)	25.2	24.3	50.6	<0.001
Protestant	66 (4.2)	15.2	34.8	50.0	
Muslim	45 (2.9)	40.0	15.6	44.4	
Other	38 (2.4)	13.2	28.9	57.9	
No Confession	272 (17.2)	11.8	15.8	72.4	
Household size					
Single	563 (35.7)	20.4	22.9	56.7	0.072
Dual	569 (36.1)	23.2	20.7	56.1	
3+ Persons	446 (28.3)	24.3	26.5	49.1	
Cared for ill					
No	1,091 (69.1)	23.3	20.7	56.0	0.003
Yes	487 (30.9)	20.9	28.5	50.5	
Cared for dying					
No	1,111 (70.4)	23.8	20.5	55.7	<0.001
Yes	467 (29.6)	19.7	29.3	51.0	
Occupation health sector					
No	1,495 (94.7)	22.7	22.5	54.8	0.060
Yes	83 83 (5.3)	20.5	33.7	45.8	
Continuous variables	N (mean ± sd)	mean (sd)	mean (sd)	mean (sd)	anova *p*-values
Age (years)	1,578 (49.7 ± 16.3)	50.1 (17.3)	51.0 (15.7)	49.0 (16.1)	0.450/0.270
Subjective Health (1–5)	1,578 (2.04 ± 0.88)	2.13 (0.84)	2.03 (0.87)	2.02 (0.90)	0.131/0.048
Religiosity (1–4)	1,578 (2.70 ± 0.89)	2.48 (0.97)	2.52 (0.82)	2.88 (0.85)	0.590/<0.001
Liberalism (1–4)	1,578 (2.22 ± 0.73)	2.57 (0.91)	2.09 (0.61)	2.15 (0.69)	<0.001/<0.001

Unweighted data. WLPT = withdrawing life-prolonging treatment, EUT = euthanasia. Reference category = non-acceptance of both WLPT and EUT. The first anova *p*-value refers to WLPT only, the second to EUT (and WLPT) in comparison to the reference category non-acceptance of both WLPT and EUT. Differences to 100 % per row are due to rounding

### Multivariate analysis

Table 2 shows the multinomial logistic regression coefficients as odds ratios (OR) between disapprovers of both WLPT and EUT (= reference category), and those who accept WLPT alone on the one hand, and those who accept EUT (and WLPT) on the other hand. As McFadden’s pseudo-R^2^ coefficients between 0.2 and 0.4 are defined as excellent model fit [33], our model fit (R^2^ = 0.077) can be considered as moderate. Unlike in the bivariate analysis, sex showed no independent statistically significant effect on the outcomes. University graduates are significantly more likely to accept WLPT (*p* > 0.001) or both EUT and WLPT (*p* = 0.005), but the effect is stronger regarding the former (OR_WLPT only_ = 4.13, OR_EUT (+WLPT)_ = 2.40) and also implied a more gradual increase across education levels only in this case. Individuals without religious confession were more likely to accept EUT (and WLPT), whereas no significant differences were found in this regard between rejecting both forms and approving solely WLPT. Using acceptance of WLPT only as reference category showed that those without religious confession had a 70 % higher chance of accepting EUT (and WLPT) instead of WLPT alone (CI = 1.17-2.55, *p* = 0.006) compared to respondents belonging to a religious confession. Furthermore, protestants (OR = 2.14, *p* = 0.063) seemed more likely than Catholics to approve of WLPT alone (compared to those rejecting both), although the effect remained statistically non-significant. Similarly, individuals from other denominations seemed more likely than their Catholic counterparts to approve of both WLPT alone, and of WLPT and EUT, but again these effects (OR_WLPT only_ = 2.36, *p* = 0.131; OR_EUT (+WLPT)_ = 2.41, *p* = 0.100) remained statistically non-significant. Unlike the results of the bivariate analysis, caring for seriously ill was not significantly related to the different outcome types. In contrast to carers for seriously ill people, respondents who had already provided care to dying individuals were, independent of other characteristics, more likely to approve of WLPT alone than those without such experience (OR = 1.69, *p* = 0.022); a weaker and non-significant effect was observed regarding acceptance of both EUT and WLPT (OR = 1.42, *p* = 0.086). In direct comparison of approving of WLPT only and approving of both WLPT and EUT, no statistically significant difference regarding care provision to dying individuals showed (*p* = 0.353). No statistically significant difference between health professionals and laypeople was found. With each decrease in the level of religiosity, the odds to approve of EUT (and WLPT) increases by 70 %, whereas the odds of approving of WLPT alone while rejecting EUT increase only by 20 % (*p* = 0.076). This also shows in the direct comparison between accepting WLPT only and WLPT and EUT as with each decrease in religiosity, respondents were more likely to accept EUT (and WLPT) instead of only WLPT by 42 % (CI = 1.19-1.67, *p* < 0.001). Finally, and in contrast to religiosity, the level of self-reported liberalism clearly differentiated between disapprovers of both end-of-life interventions and approvers of WLPT alone or approvers of EUT (and WLPT). With each increase in the level of liberalism, the chances to approve of WLPT alone or both EUT and WLPT more than doubled compared to the reference category, i.e. to individuals rejecting both WLPT and EUT. This is also reflected in the direct comparison when WLPT only is used as reference category, as no statistically significant difference (*p* = 0.634) showed regarding liberalism between respondents who accepted WLPT only and those who accepted both WLPT and EUT.

**Table 2:** Multinomial logistic regression analysis

	Acceptance WLPT only		Acceptance WLPT & EUT	
	OR (CI)	*p*-value	OR (CI)	*p*-value
Socio-demographics				
Gender (male = ref)				
Female	0.91 (0.66-1.25)	0.547	0.82 (0.62-1.08)	0.161
Age	1.01 (1.00-1.02)	0.069	1.01 (1.00-1.02)	0.252
Education (compulsory school = ref)				
Apprentice/vocational	1.58 (0.98-2.53)	0.059	1.36 (0.92-2.00)	0.123
High school diploma	1.72 (0.95-3.12)	0.073	1.04 (0.95-3.12)	0.889
University	4.13 (2.11-8.09)	<0.001	2.40 (1.31-4.39)	0.005
Confession (Catholic = ref)				
Protestant	2.14 (0.96-4.78)	0.063	1.45 (0.67-3.11)	0.344
Muslim	0.47 (0.19-1.20)	0.117	0.66 (0.32-1.34)	0.251
Other	2.36 (0.77-7.22)	0.131	2.41 (0.85-6.85)	0.100
No Confession	1.04 (0.61-1.77)	0.856	1.82 (1.17-2.82)	0.008
Household size (single = ref)				
Dual	0.79 (0.54-1.15)	0.216	0.93 (0.68-1.27)	0.636
3+ persons	0.94 (0.64-1.40)	0.775	0.73 (0.51-1.02)	0.067
Health status	0.92 (0.76-1.12)	0.403	0.92 (0.77-1.08)	0.309
Personal experience				
Cared for ill (no = ref)				
Yes	1.00 (0.64-1.57)	0.991	0.95 (0.63-1.41)	0.786
Cared for dying (no = ref)				
Yes	1.69 (1.08-2.66)	0.022	1.42 (0.95-2.13)	0.086
Occupation health sector (no = ref)				
Yes	1.36 (0.68-2.70)	0.390	0.94 (0.49-1.80)	0.848
Orientations				
Religiosity	1.20 (0.98-1.48)	0.076	1.69 (1.42-2.01)	<0.001
Liberalism	0.45 (0.36-0.57)	<0.001	0.49 (0.41-0.59)	<0.001
Intercept	0.37 (0.16-0.85)	0.020	1.60 (0.80-3.19)	0.183
N	1,578			
Log-likelihood	1,466			
R^2^ (Mc Fadden)	0.077			

Unweighted data. WLPT = withdrawing life-prolonging treatment, EUT = euthanasia. Reference category = non-acceptance of both WLPT and EUT. OR = odds ratio, CI = 95-% confidence interval

## Discussion

In a nationally representative CAPI survey, we asked respondents to approve or decline of end-of-life interventions in case of terminally ill and heavily suffering individuals, who request either WLPT or EUT. Less than one quarter of the respondents rejected both WLPT and EUT, or approved solely WLPT but still rejected EUT, whereas more than half of the Austrian population approved both end-of-life interventions in the aforementioned case. The fact that a considerable number of respondents approved WLPT but not EUT implies that withdrawal or limiting treatment and ending of life actively by physicians are recognised clearly as different kinds of end-of-life interventions in the general population, although a majority approves of both. These results are compatible with previous studies from other countries regarding differences in the prevalence of approval between WLPT and EUT [11, 12, 14, 15, 18]. In contrast to previous studies we linked popular acceptance for both end-of-life interventions in order to distinguish determinants for individuals who rejected both WLPT and EUT, approved WLPT alone, or WLPT *and* EUT. In this regard, no previously implied gender-differences [14, 15] were found. This could be due to the greater number of predictor variables controlled for in our study, in particular care-giving, confession and religiosity. Whereas caring for seriously ill individuals did not have an effect for the approval of WLPT and EUT, a difference was found in respondents with experience in caregiving for dying individuals. This experience led respondents to more likely change from rejecting both types of end-of-life interventions to approve WLPT (alone). This could be attributable to witnessing suffering persons dying. However, since we do not have further details for these care-giving settings, the underlying mechanism remains unclear. Unlike previous studies [14, 15], we found no difference in attitude between laypersons and health professionals. This could be due to the small number of physicians included in the sample, i.e. due to the numerical dominance of nurses and other health professionals. These groups, in contrast to physicians, had previously reported high levels of approval [34]. Similar to [25], we found a strong difference between individuals with a low (compulsory school) and those with a very high (university degree) level of education, meaning that the latter approved of WLPT and EUT to a higher extent. This polarising effect between individuals with the lowest and highest educational level was stronger when it came to the approval of WLPT alone, as opposed to approval of both types of end-of-life interventions. In contrast to previous unclear findings on education in this regard [14, 15], this means that independent of religiosity and liberalism, higher education seems particularly associated with approval of withdrawing live-prolonging treatment but less strong associated with euthanasia. This could be due the increased awareness among higher educated that the historical charged term of “euthanasia” was used also to describe the in-voluntary killing of disabled children and adults during the Nazi regime in Austria (1938–1945). The differing attitudes towards WLPT and EUT regarding religion have already been outlined by previous studies [14, 18]. Although the effect sizes were substantial, we could not find statistically significant differences regarding acceptance of end-of-life interventions between religious confessions. This statistically non-significant more liberal stance of Protestants and other religious denominations in comparison to the Catholics – which comprise the largest religious group in Austria – might be due to the small n (Protestants: *n* = 66, other denominations: *n* = 38) and/or heterogeneity across different sub-denominations among the umbrella terms ‘Protestant’ and ‘other denominations’. The level of religiosity and being without confession strongly impacts acceptance of euthanasia EUT but is not relevant regarding withholding of live-prolonging treatment alone, which confirms findings from the two other existing studies in this regard [14, 15]. It seems therefore, that the sanctity of life argument applies only to the deliberate and requested ending of a patient’s life by a medical doctor but not, as expected, to withholding life-prolonging treatment. Religious and non-religious people similarly approve to not artificially prolong the life of terminally ill and suffering patients if requested by the patient. Much like previous studies, we were able to confirm the finding that the level of liberalism acts as an important socio-cultural orientation regarding end-of-life interventions [21, 24–26]. Socio-cultural liberalism had a stronger impact than religion regarding the attitude towards euthanasia, and in contrast to religion, showed almost identical effects for WLPT and EUT. This could mean, that unlike religious people, liberal individuals highly value personal autonomy and freedom regarding life and death, irrespective of the legal, technical or ethical differences between WLPT and EUT, as long as both types of end-of-life interventions are patient requested.

### Strengths & limitations

Based on a large and representative sample of the Austrian adult population, this study provided results on the acceptance of end-of-life interventions, distinguishing between rejecting both WLPT and EUT, accepting solely WLPT, and accepting both WLPT and EUT. The scope of the study is limited by the cross-sectional nature of the survey data, which does not allow causal inferences, the exploratory character of the study, and the use of self-rating questions regarding orientations instead of scales. Furthermore, not all factors potentially relevant could be included in the analyses, among them in particular attitudes regarding age and ageing, trust in the health care system, and experiences with end-of-life-care and death of close relatives. These determinants will require future research.

## Conclusion

This study examined the possible differences between acceptance of end-of-life interventions – none, withdrawing life-prolonging treatment only, and euthanasia (and life-prolonging treatments) – in the general population of Austria and assessed differences in the determinants. The representative survey data showed, that higher education and stronger socio-cultural liberal orientations increased the likelihood of approving of both withdrawing of life-prolonging treatment and euthanasia, whereas personal experience with end-of-life care increased the likelihood of approval of the former only, and religiosity decreased the approval of euthanasia only. In conclusion, this study found evidence for both the same (education, liberalism) and different (religiosity, care experiences) determinants for the acceptance of withdrawing life-prolonging treatment and euthanasia.
